# Diagnosis of Extra-Skeletal Ewing Sarcoma in an 11-Year-Old Child with Large Thoracic Mass revealed by [^18^F]FDG PET/CT

**DOI:** 10.1007/s13139-025-00964-8

**Published:** 2025-12-09

**Authors:** Florian Rosar, Moritz B. Bastian, Caroline Burgard, Marc Remke, Stéphane Collaud, Hafsa Kaman, Samer Ezziddin, Dominik Schöndorf

**Affiliations:** 1 Department of Nuclear Medicine, Saarland University, Kirrberger Str., Geb. 50, 66421 Homburg, Germany; 2 Department of Pediatric Oncology and Hematology, Saarland University, Homburg, Germany; 3 Department of Thoracic Surgery, Cologne-Merheim Hospital, University of Witten/Herdecke, Cologne, Germany

An 11-year-old girl was admitted as an emergency with severe dyspnea that started that morning. Examination revealed absent breath sounds on the left side. A chest X-ray, CT and MRI of the thorax revealed a left-sided giant thoracic mass, highly suspicious for malignancy. Retrospectively, unspecific symptoms mimicking recurrent respiratory infections began two months earlier with cough, fever, and weakness and were treated once with antibiotics. Dyspnea developed one week before admission and worsened despite anti-obstructive inhalation therapy. ^18^F-fluorodeoxyglucose ([^18^F]FDG) positron emission tomography/computed tomography (PET/CT) scan (acquisition 60 min post injection of 152 MBq) was performed for staging (Fig. 1), showing the mass with inhomogeneous, mostly intense [^18^F]FDG-uptake (SUVmax: 11.3 and SUVmean: 5.5). A voluminous mass, approximately 15 × 15 × 30 cm in size, filled the entire left hemithorax (without bone involvement), causing rightward displacement of the mediastinum. No other suspicious findings were observed by [^18^F]FDG PET/CT, in accordance with a negative MRI and negative bone-marrow biopsies.

**Fig. 1:**
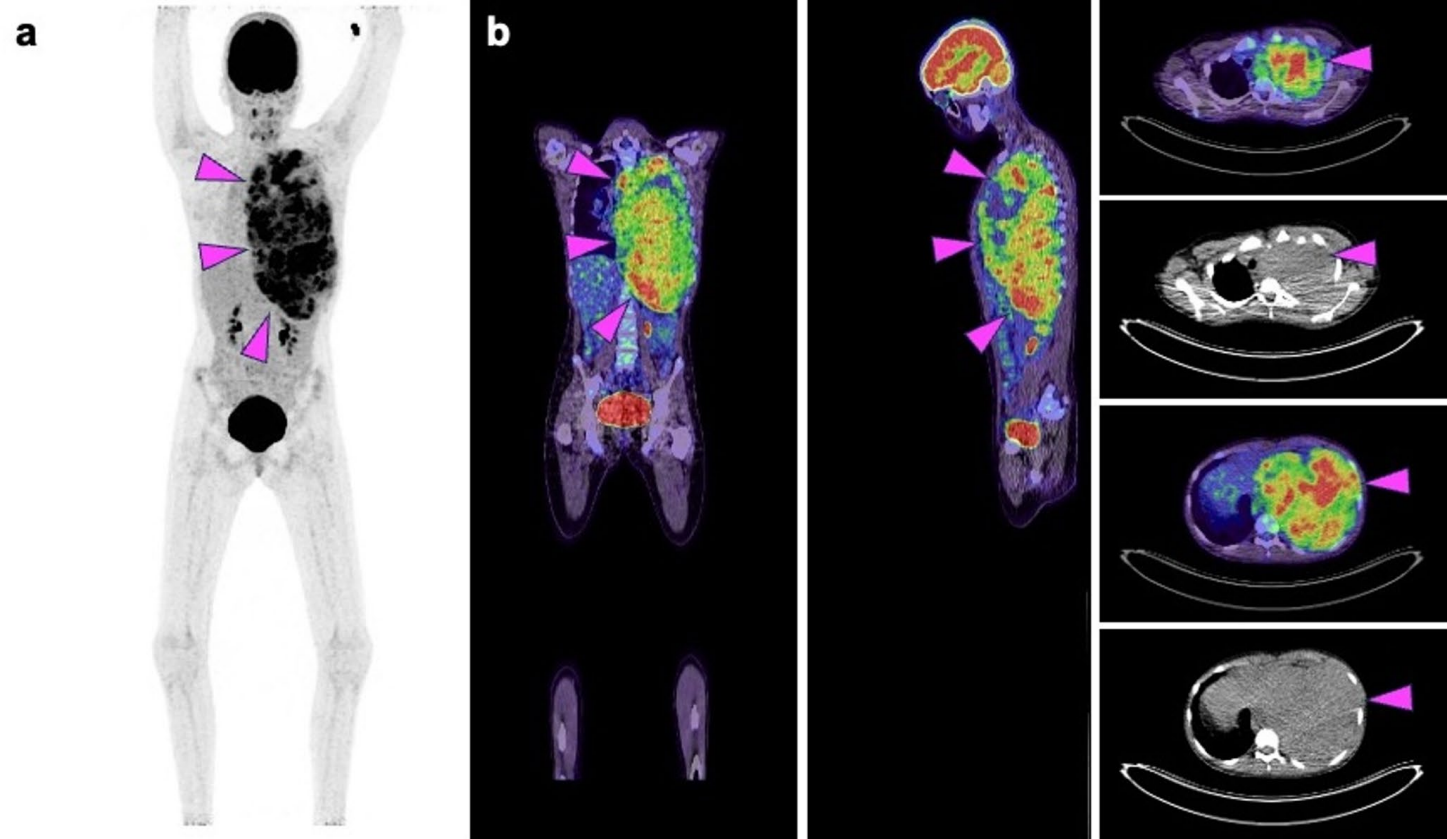
[^18^F]FDG PET/CT scan of an 11-year-old girl with a primary pleural extra-skeletal Ewing sarcoma (EwS). **a**: Maximum intensity projection (MIP) and **b**: exemplary transversal, sagittal and coronal slices of [^18^F]FDG PET/CT fusion (or corresponding low-dose CT) showing the mass with inhomogeneous, mostly intense [^18^F]FDG uptake (SUVmax: 11.3 and SUVmean: 5.5). The giant mass, measuring approximately 15 × 15 × 30 cm, occupied the entire left hemithorax without rib/bone involvement, and displacing the mediastinum to the right (magenta arrows)

The main differential diagnosis included Ewing sarcoma (EwS), other sarcomas, malignant lymphoma, neuroblastoma, germ cell tumor or other solid tumors. The mass was subsequently biopsied under MRI-guidance.

Histopathological examination with immunohistochemical staining confirmed CD-99-positive EwS, supported by an EWSR1 translocation (22p12). Thus, the final diagnosis was a localized extra-skeletal EwS. The patient underwent neoadjuvant chemotherapy, followed by pleurectomy/decortication combined with hyperthermic intrathoracic chemotherapy, further adjuvant chemotherapy, and radiation therapy. Following this treatment regimen, the child is now in remission.

EwS is the second-most prevalent bone tumor among children and adolescents [1]. It is an aggressive malignancy with a poor prognosis, but systemic treatment has significantly improved patient outcomes for localized disease. Disease extent, tumor size and tumor location are strongly associated with survival. Extra-skeletal EwS accounts for about 20–30% of EwS [2], and the management of thoracic EwS remains a challenge. [^18^F]FDG PET/CT can be used for diagnosing, staging, and detecting metastases or recurrence of EwS [3]. [^18^F]FDG PET/CT can also serve as a prognostic tool to predict survival outcomes in EwS [4]. Although [^18^F]FDG PET/CT is commonly used for EwS, cases of extra-skeletal EwS presenting as a large, highly metabolically active mass occupying the entire left hemithorax are rarely reported in literature; more commonly, the reported cases involve less extensive disease [5].

This striking image serves as a reminder to clinicians to consider extra-skeletal EwS in the differential diagnosis of patients, particularly children, presenting with a large thoracic mass, without rib/bone involvement.

## Data Availability

Data sharing is not applicable to this article as no datasets were generated or analyzed during the current study.
